# Recombinant thrombomodulin may protect cardiac capillary endothelial glycocalyx through promoting Glypican-1 expression under experimental endotoxemia

**DOI:** 10.1016/j.heliyon.2022.e11262

**Published:** 2022-10-25

**Authors:** Yoshinori Kakino, Tomoaki Doi, Hideshi Okada, Kodai Suzuki, Chihiro Takada, Hiroyuki Tomita, Hirotaka Asano, Soichiro Kano, Yugo Wakayama, Tomoki Okuda, Hirotsugu Fukuda, Ayane Nishio, Yuki Kawasaki, Ayumi Kuroda, Takuto Shimada, Shigeo Takashima, Keiko Suzuki, Genki Yoshimura, Ryo Kamidani, Ryu Yasuda, Tetsuya Fukuta, Yuichiro Kitagawa, Haruka Okamoto, Takahito Miyake, Akio Suzuki, Takahiro Yoshida, Nobuyuki Tetsuka, Shozo Yoshida, Shinji Ogura

**Affiliations:** a Department of Emergency and Disaster Medicine, Gifu University Graduate School of Medicine, Gifu, Japan; b Department of Tumor Pathology, Gifu University Graduate School of Medicine, Gifu, Japan; c Division of Genomics Research, Life Science Research Center, Gifu University, Gifu, Japan; d Department of Pharmacy, Gifu University Hospital, Gifu, Japan; e Department of Infection Control, Gifu University Graduate School of Medicine, Gifu, Japan; f Abuse Prevention Center, Gifu University Graduate School of Medicine, Gifu, Japan

**Keywords:** Vascular endothelium, Glycocalyx, Microvascular dysfunction, Thrombomodulin, Glypican-1

## Abstract

**Introduction:**

Myocardial dysfunction occurs in patients with sepsis due to vascular endothelial injury. Recombinant human thrombomodulin (rhTM) attenuates vascular endothelial injuries through endothelial glycocalyx (eGC) protection.

**Hypothesis:**

We hypothesized that rhTM attenuates myocardial dysfunction via the inhibition of vascular endothelial injury during sepsis.

**Methods:**

Ten-week-old male C57BL6 mice were injected intraperitoneally with 20 mg/kg of lipopolysaccharide (LPS). In rhTM-treated mice, rhTM was injected intraperitoneally at 3 and 24 h after LPS injection. Saline was injected intraperitoneally as control. To assess for eGC injury, intensity score was measured 48 h after the LPS injection. To confirm vascular endothelial injuries, ultrastructural analysis was performed using scanning (SEM) and transmission electron microscopy (TEM).

**Results:**

The survival rate of the rhTM group at 48 h after LPS injection was significantly higher than that of the control group (68% vs. 17%, p < 0.05). The serum level of troponin I in the rhTM group was lower than that in the control (2.2 ± 0.4 ng/dL vs 9.4 ± 1.1 ng/dL, p < 0.05). The expression of interleukin-6 (IL-6) was attenuated in the rhTM-treated group than in the control (65.3 ± 15.3 ng/mL vs 226.3 ± 19.4 ng/mL, p < 0.05). The serum concentration of syndecan-1, a marker of glycocalyx damage, was significantly decreased 48 h post-administration of LPS in the rhTM-treated group than in the control group. In ultrastructural analysis using SEM and TEM, eGC peeled off from the surface of the capillary lumen in the control. Conversely, the eGC injury was attenuated in the rhTM group. Gene set enrichment analysis revealed that osteomodulin, osteoglycin proline/arginine-rich end leucine-rich repeat protein, and glypican-1, which are proteoglycans, were preserved by rhTM treatment. Their protein expression was retained in endothelial cells.

**Conclusion:**

rhTM attenuates sepsis-induced myocardial dysfunction via eGC protection.

## 1 Introduction

Sepsis is an auto-destructive process in which excessive immune responses to invading microorganisms result in organ dysfunction. In particular, the mortality rate for septic shock, the most severe form of sepsis, is approximately 40%, despite recent advances in intensive care [1]. Organ failure, including myocardial damage, is one of the diagnostic criteria for sepsis [2]. Septic myocardial failure and elevated troponin are often observed in septic patients [3], and those with impaired cardiac function are known to have a higher overall mortality rate than those without impaired cardiac function [4]. Myocardial failure is considered one of the most important factors in the prognosis of sepsis. Indeed, myocardial microcirculatory impairment is caused by the narrowing of myocardial cells and edema of the capillary walls and capillary lumen [5]. These findings suggest that septic myocardial injury results from microvascular damage and that sepsis-induced cardiac dysfunction is derived from microvascular endothelial damage and is associated with damage to vascular endothelial glycocalyx [6].

Healthy endothelial cells are covered with glycocalyx, a glycoprotein [7, 8, 9], which plays a role in maintaining vascular homeostasis [10, 11, 12, 13]. However, in septic conditions, the endothelial glycocalyx is damaged by several inflammatory cytokines, resulting in organ failure. Cytokines impair the thickness and stiffness of the endothelial glycocalyx using lipopolysaccharide (LPS) [14, 15, 16].

Thrombomodulin expressed on the surface of healthy endothelial cells maintains vascular homeostasis [17]. It has anti-inflammatory effects via binding to high-mobility group box 1 (HMGB1), a nuclear architectural chromatin-binding protein involved in DNA organization and transcription regulation [18]. HMGB-1 plays a crucial role in ARDS progression [19, 20, 21]. Recombinant human soluble thrombomodulin (rhTM) has beneficial treatment effects for patients with disseminated intravascular coagulation (DIC) [18]. rhTM upregulates the TGF-β signaling and JAK-STAT pathways, contributing to cell proliferation, survival, and differentiation [22]. Moreover, rhTM upregulated the heparan sulfate 6-O-sulfotransferase 1 gene, which is essential for heparan sulfate synthesis, a component of the endothelial glycocalyx [22].

However, the effects of rhTM on sepsis-induced myocardial injury have not been investigated. Therefore, we aimed to determine the condition of myocardial endothelial glycocalyx after LPS administration in mice treated with rhTM.

## 2 Methods

### 2.1 In vivo animal studies

This study was conducted in accordance with the Care and Use of Laboratory Animals Guide published by the National Institutes of Health (NIH publication, 8th Edition, 2011) and was approved by the Institutional Animal Research Committee of the Gifu University, Gifu, Japan. Ten-week-old Male C57BL6 mice were procured from Chubu Chemical Materials Co. (Nagoya, Japan). After 16 h of starvation, LPS (20 mg/kg; MilliporeSigma, Burlington, MA, USA) was injected intraperitoneally, and recombinant human thrombomodulin (rhTM, 30 mg/kg; Asahi Kasei Pharma Corporation, Tokyo, Japan) dissolved in 100 μL saline was injected at 3 h and 24 h after LPS injection, survival was measured at 12, 24, 36, and 48 h after LPS administration. As controls, we prepared mice that received the same volume of saline after LPS administration (Control) and mice that received neither LPS nor rhTM (Sham). Surviving mice were operated on, and the heart samples were collected.

### 2.2 Serum Preparation and enzyme-linked immunosorbent assay (ELISA)

Blood samples were collected from the maxillary artery, allowed to clot at room temperature for 2 h, and centrifuged at 2,000×*g* at 4 °C for 20 min. Blood samples were obtained on six independent samples from individual mice from each group. The supernatant was used to measure the serum IL-6, troponin-I, and syndecan-1 levels using ELISA Quantitation Kits for mouse IL-6 (cat no. M6000B; R&D Systems, Inc.), cardiac troponin-I (CTNI-1-HS; Life Diagnostics, Inc., West Chester, PA, USA), and syndecan-1 (Cat. No. 860.090.192; Diaclone, Besancon Cedex, France).

### 2.3 Scoring of lectin-staining intensity

For the quantitative analysis of glycocalyx injury in the heart, the scoring of wheat germ agglutinin (WGA, B-1025-5; Vector Laboratories, Burlingame, CA, USA) staining intensity was performed using a fluorescence microscope (BZ-X810, Keyence Corp., Osaka, Japan) and ImageJ software. Samples were prepared, and the intensity score was measured as previously described [22]. The intensity of WGA was scored manually in ten high-power fields per sample (n = 6 per sample) in the focal plane.

### 2.4 Microarray analysis

For microarray analysis, cardiac tissues were collected from the saline- and rhTM-treated mice 30 h after LPS administration (n = 3 each). The total RNA was extracted using a Maxwell RSC instrument with a simple RNA tissue kit (Promega, Fitchburg, WI, USA). Gene expression analysis of the RNA samples was performed at the Gifu University Research Center for Life Science (Gifu, Japan) using an Agilent Expression Array (SurePrint G3 Mouse GE 8 × 60 K Microarray). Genes differentially expressed between the rhTM-treated and control groups were identified by a fold change greater than or equal to 2 (upregulated) or less than or equal to 0.5 (down-regulated), and p-values ≤ 0.01 were identified. Gene set enrichment analysis (GSEA) was used to analyze pathway enrichment (http://software.broadinstitute.org/gsea/index.jsp). All the microarray data were deposited in the Gene Expression Omnibus (GEO) under accession number GSE126725 (http://www.ncbi.nlm.nih.gov/geo/).

### 2.5 Immunohistochemistry

After deparaffinization, 4-μm-thick sections of the heart were cut and incubated with primary antibodies against glypican-1 (GPC-1, ab217339; Abcam, Cambridge, UK). The target proteins were visualized using the VECTASTAIN Elite ABC system (Vector Laboratories). To further confirm their location, double fluorescence staining was performed using frozen sections. WGA lectin labeled with fluorescent dye was injected into the jugular vein directly to avoid non-specific staining. Endothelial glycocalyx was visualized. The mice were killed within 10 min after the WGA injection, and frozen sections were prepared using the primary antibodies GPC-1. The target proteins were visualized using secondary antibodies (Alexa Fluor 488, Invitrogen) and Hoechst nuclear staining. Immunohistochemistry was performed on six independent samples from individual mice from each group.

### 2.6 Electron microscopy

Electron microscopic analysis of the endothelial glycocalyx of the heart was performed as described previously [16]. Briefly, the mice were anesthetized and then perfused with a solution composed of 2% glutaraldehyde, 2% sucrose, 0.1 M sodium cacodylate buffer (pH 7.3), and 2% lanthanum nitrate, at a steady flow rate of 1 mL/min, through a cannula placed in the left ventricle. After sacrificing the mice, the heart samples were fixed in a solution without glutaraldehyde and washed thereafter in an alkaline (0.03 M NaOH) 2% sucrose solution. The freeze-fracture method was used to prepare the samples for scanning electron microscopy (SEM; S-4800, Hitachi, Tokyo, Japan). The method for transmission electron microscopy (TEM) included embedding the specimens in epoxy resin. This was followed by the generation of ultrathin (90-nm) sections stained with uranyl acetate and lead citrate and subjected to TEM analysis (HT-7700, Hitachi). To prepare the samples for conventional electron microscopy, 2.5% glutaraldehyde in 0.1 M phosphate buffer (pH 7.4) without lanthanum nitrate was used as the fixative. Electron microscopy was performed on three independent samples from individual mice from each group.

### 2.7 Statistical analysis

Data are presented as mean ± SEM. The Student's two-tailed *t*-test was used to compare the two groups, and survival data were analyzed using the log-rank test. *P* values <0.05 were considered statistically significant. All calculations were performed using the Prism software version 7.02 (GraphPad, La Jolla, CA, USA).

## 3 Results

**Figure 1:**
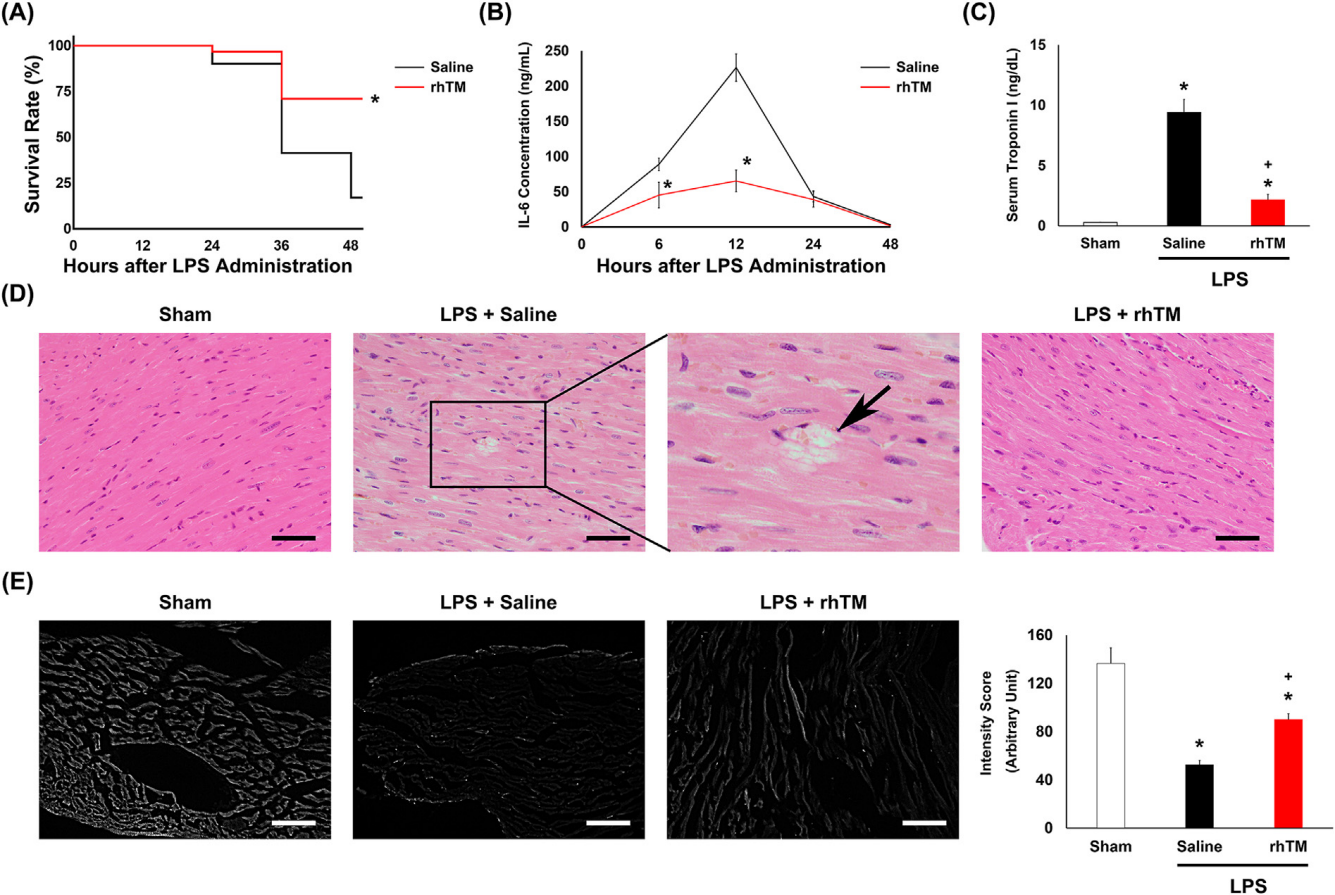
**The effects of recombinant human thrombomodulin (rhTM) administration on LPS-induced myocardial injury.** (A) Kaplan-Meier survival curves of saline-treated (n = 60) and rhTM-treated (n = 41) mice after LPS administration. ∗P < 0.05 versus saline-treated mice. (B) Serum IL-6 and (C) troponin I concentrations were measured in the mice using ELISA (n = 6 each). ∗P < 0.05 versus saline-treated mice (B). P < 0.05 versus sham mice. + P < 0.05 versus saline-treated mice after LPS administrated (C). (D) Hematoxylin and eosin-stained heart tissues; the arrow indicates a site of rupture and necrosis of the cardiomyocytes. Bars: 50 μm. (E, F) WGA intensity score measurement. (E) Lectin, a glycocalyx-binding glycoprotein, stained by wheat germ agglutinin (WGA). Bars: 50 μm. (F) The graph of WGA intensity of sham-, saline- or rhTM-treated mice after LPS administration mice. ∗P < 0.05 versus sham mice. + P < 0.05, versus saline-treated mice after LPS-administration.

To create an endotoxemia model, 10-week-old C57BL/6 male mice were intraperitoneally injected with LPS. The survival rate of rhTM-treated mice (69%, 28/41) was significantly higher than that of the saline-injected group (17%, 10/60) 48 h after LPS administration (Figure 1A).

The proinflammatory cytokine and IL-6 in plasma increased after 6 h of LPS administration (89.0 ± 9.0 ng/mL) and reached the peak at 12 h (226.3 ± 19.4 ng/mL) after LPS administration. Conversely, the IL-6 concentration was significantly reduced in the rhTM-treated mice than in the saline-injected mice at 6 h and 12 h after LPS injection (45.2 ± 18.3 ng/mL, 65.3 ± 15.3 ng/mL, respectively) (Figure 1B). Serum troponin I level in the rhTM-treated mice after LPS injection were significantly lower than those in the saline-treated group (2.2 ± 0.4 ng/dL vs 9.4 ± 1.1 ng/dL, p < 0.05, Figure 1C).

Histological analysis was performed. Although the cardiomyocytes were intact and regular in the sham mice, necrosis was observed in the LPS mice. There was little damage to the rhTM-treated hearts 48 h after LPS administration (Figure 1D). After LPS administration, the capillaries were broken in the heart (SupplementaryFigure).

For the quantitative assessment of endothelial glycocalyx injury, intensity scoring was performed using WGA lectin, which binds with glycoproteins within the endothelial glycocalyx [23] (Figure 1E). The endothelial glycocalyx was partially restored to normal intensity in rhTM-treated mice. This result suggests that rhTM treatment inhibited LPS-induced endothelial glycocalyx injury in the heart.

### 3.1 Ultrastructure of endothelial glycocalyx injuries

Although the quantification of glycocalyx injury by lectins is very useful, it is difficult to confirm the structure of endothelial glycocalyx. Therefore, electron microscopy analysis was performed using lanthanum staining for glycocalyx visualization.

**Figure 2:**
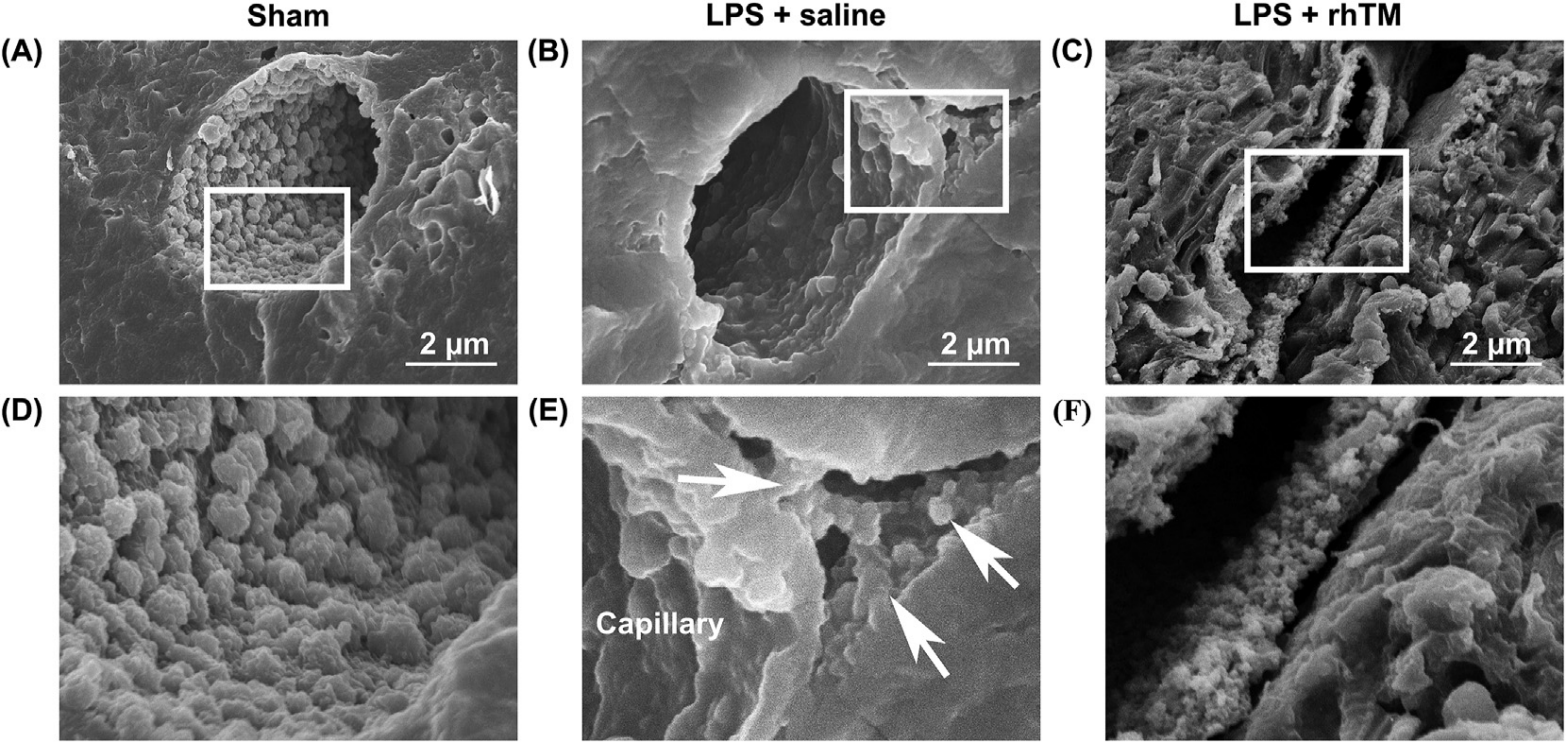
**Ultrastructure of cardiac capillaries in hearts after LPS administration using a scanning electron microscope.** (A–C) Scanning electron microscope images. The endothelial glycocalyx was detected using lanthanum nitrate staining. Bars: 2 μm. (D–F) were expanded images in white squares in (A–C), respectively. The white arrows indicated lanthanum nitrate particles out of the capillary.

Although the endothelial glycocalyx covered the surface of cardiac capillaries in sham mice hearts (Figure 2A and D), the endothelial glycocalyx was degraded after LPS injection exposing the surface of the endothelial cells to the vascular lumen (Figure 2B). The glycocalyx was visualized out of the capillaries and interstitials using lanthanum nitrate (Figure 2E). Conversely, the endothelial glycocalyx injury was attenuated, and the glycocalyx structure remained on the surface of the endothelium in the rhTM-treated hearts (Figure 2C). In addition, there was little interstitial endothelial glycocalyx in the rhTM-treated hearts (Figure 2F).

**Figure 3:**
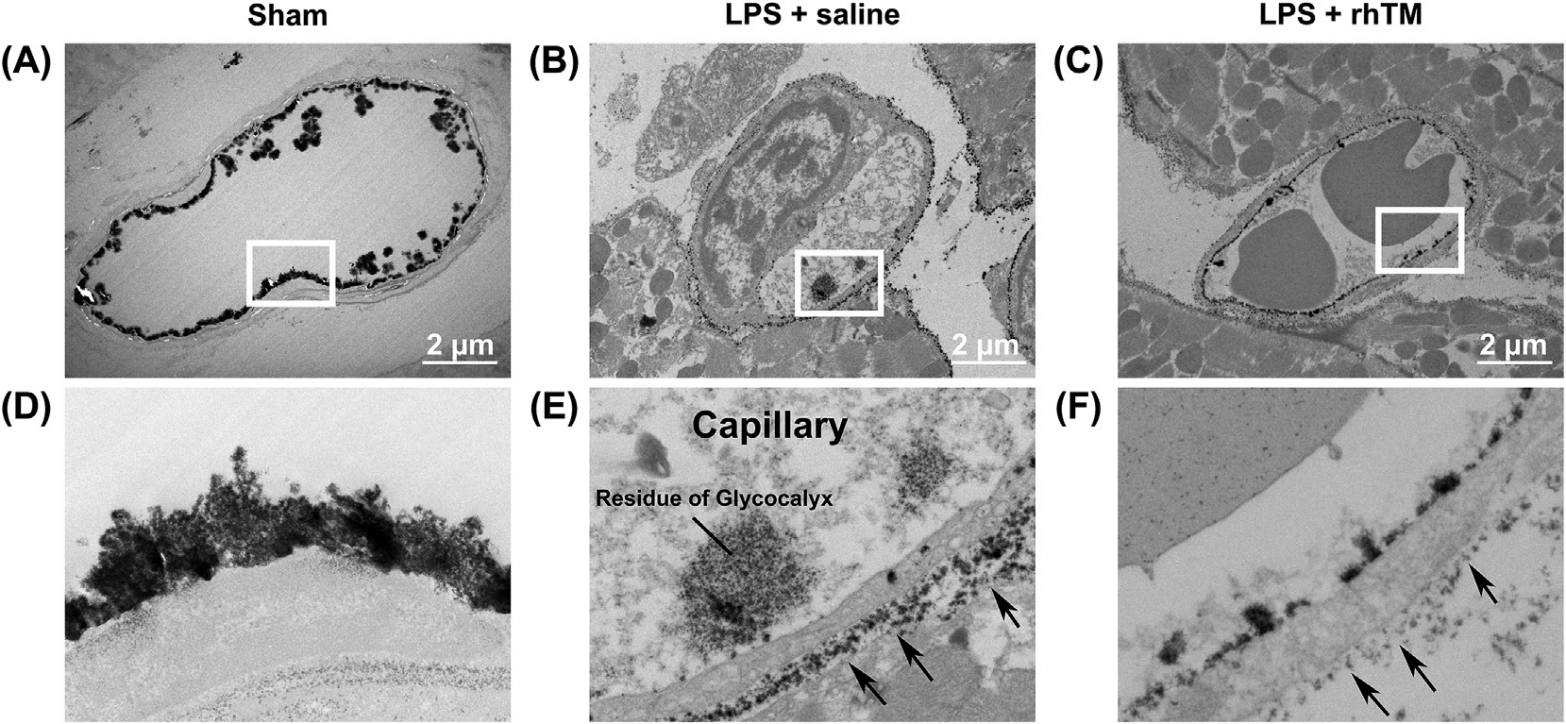
**Ultrastructure of cardiac capillaries in hearts after LPS administration using a transmitted electron microscope.** (A–C) Transmitted electron microscope images. The endothelial glycocalyx was detected using lanthanum nitrate staining. Bars: 2 μm. (D–F) were expanded images in white squares in (A–C), respectively. The black arrows indicated lanthanum nitrate particles out of the capillary.

Transmitted ultrastructural microscopy observations were performed to determine the detailed positional relationship between them. In sham mouse hearts, a thick endothelial glycocalyx exists on the surface of the endothelial cells, similar to the SEM imaging (Figure 3A and D). LPS administration injured the endothelial glycocalyx, and little endothelial glycocalyx existed on the surface of the endothelium (Figure 3B). While the remaining endothelial glycocalyx was in the vascular lumen, lanthanum nitrate particles were found interstitially (Figure 3E). Endothelial glycocalyx injury was inhibited by the rhTM treatment (Figure 3C), and the structures remained in the vascular endothelium (Figure 3F). In addition, the efflux of lanthanum nitrate particles from outside the capillary was inhibited compared with the saline-treated mice (Figure 3F).

### 3.2 Gene set enrichment analysis following rhTM treatment of the hearts

**Figure 4:**
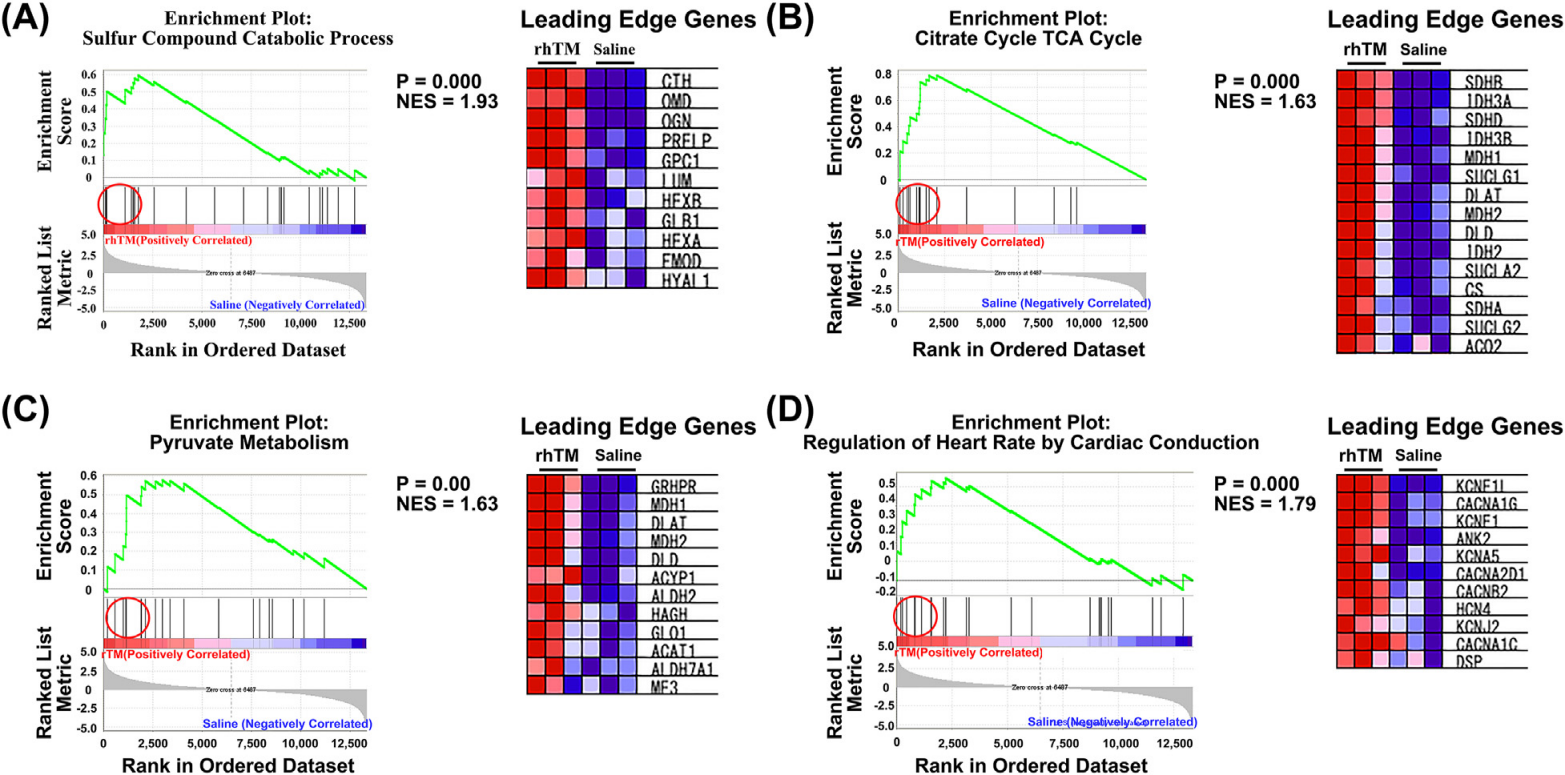
**Gene set enrichment analysis.** (A–D) Enrichment plots and leading-edge gene lists. (A) Sulfur compound catabolic process, (B) Citrate cycle TCA cycle, (C) Pyruvate metabolism, and (D) Regulation of the heart rate by cardiac conduction. NES, normalized enrichment score.

A comprehensive analysis was performed to determine which genes in the heart were affected by the rhTM treatment in LPS-induced vasculitis. GSEA was performed for the saline- and rhTM-treated groups. Gene ontology (GO) and Kyoto Encyclopedia of Genes and Genomes (KEGG) analyses of GSEA, gene sets associated with sulfur compound catabolic process (Figure 4A), citrate cycle TCA cycle (Figure 4 B), pyruvate metabolism (Figure 4C), and the regulation of heart rate by cardiac conduction (Figure 4D) were significantly upregulated in the rhTM-treated mice than in the saline-treated mice (P < 0.01).

### 3.3 GPC1 expression in cardiac capillaries

**Figure 5:**
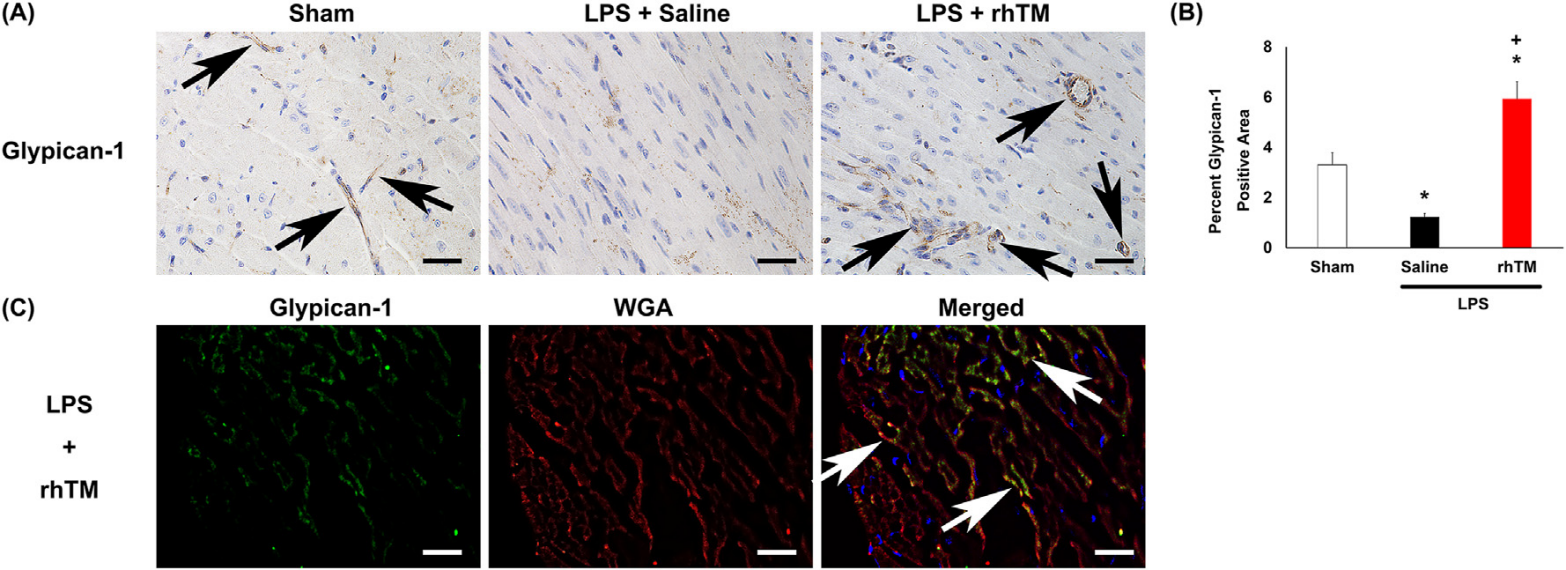
**Immunohistochemical analysis of Glypican-1** (A) Immunohistochemical analysis of Glypican-1 in the hearts of sham mice and saline-injected and recombinant human thrombomodulin (rhTM)-treated mice 48 h after LPS administration. Arrows indicate glypican-1-positive cells. (B) Graph showing glypican-1-positive positive area (n = 6 each). ∗P < 0.05 versus sham mice. + P < 0.05 versus saline-treated mice after LPS administration. (C) Immunofluorescence analysis of glypican-1 and WGA lectin. White arrows indicate co-localization of glypican-1 and WGA lectin.

In the sulfur compound catabolic process gene set, glypican-1 was promoted by rhTM treatment. Immunohistochemical analysis was performed to determine the histological distribution of glypican expression. GPC-1 was expressed in normal capillaries (Figure 5A). Although its expression was attenuated by LPS administration, rhTM treatment restored GPC expression (Figure 5B). To provide further confirmation, double immunostaining for GPC-1 and WGA lectins was performed. To visualize the capillary endothelial glycocalyx, WGA lectin labeled with a fluorescent dye was injected into the jugular vein to avoid non-specific staining. GPC-1 and WGA lectin were co-localized in rhTM-treated mice (Figure 5C). These results suggest that rhTM treatment increases glycocalyx synthesis promoted by GPC-1 expression.

## 4 Discussion

Although septic cardiomyopathy is characterized by (1) left ventricular dilatation with normal- or low-filling pressure, (2) reduced ventricular contractility, and (3) right or left ventricular dysfunction with a reduced response to volume infusion, the consistent diagnostic criteria of septic cardiomyopathy are still lacking, and the exact pathophysiologic mechanism is yet to be fully understood [24]. Therefore, including potential septic myocardial injury may be more common.

The vascular endothelial glycocalyx is present on the surface of healthy vascular endothelial cells and plays an important role in maintaining vascular homeostasis [7, 8, 9, 10, 12, 13, 16]. Intact glycocalyx protects against endothelial damage [25, 26, 27, 28]. rhTM administration affects inflammation, cell proliferation and differentiation, and glycan synthesis in the lungs and suppresses ARDS caused by endothelial injury [22]. In fact, the present study confirmed that rhTM improves survival rate and attenuates inflammatory cytokine IL6 expression. These results are consistent with our previous study [24]. Moreover, we found that rhTM has a protective effect on cardiac endothelial glycocalyx in mice after LPS administration.

The mechanism is thought to promote proteoglycan synthesis, which constitutes the glycocalyx, and the inhibition of glycocalyx degradation through its anti-inflammatory effects.

### 4.1 rhTM May improve aerobic metabolism in the heart

In rhTM-treated mice hearts, the gene sets for the TCA cycle and pyruvate metabolism were upregulated. The upregulation of these gene sets indicates that aerobic metabolism is enhanced in the heart, affecting the gene set that regulates the heart rate and cardiac conduction. The current study showed that rhTM maintained the structure of the vascular endothelial glycocalyx. Presumably, the maintenance of the vascular structure suppresses microcirculatory damage in the rhTM-treated group. Additionally, the protection of the vascular endothelial glycocalyx may have contributed.

A recent report showed that microcirculation and endothelial glycocalyx do not face concordant changes in the sepsis context [29]. However, since the morphology, components, and injury patterns of endothelial glycocalyx vary with organ and vessel morphology and type of invasion [30], it may still be controversial to conclude that microcirculation and endothelial glycocalyx do not face changes consistent with the parameters of conventional microcirculatory injury.

### 4.2 rhTM attenuated endothelial glycocalyx injury

Hypercytokinemia caused by excessive inflammation activates the complement system and overly potentiates the action of neutrophils. Elastase, released by neutrophils, damages the vascular endothelial glycocalyx. This is one of the mechanisms causing organ damage [6, 31]. We found that rhTM treatment suppressed serum IL-6 levels, which may be one of the mechanisms for the suppression of endothelial glycocalyx injury.

This study used lanthanum nitrate to visualize the vascular endothelial glycocalyx. Since the samples were fixed by reflux fixation, if the tissue were normal, lanthanum would remain in the blood vessels, and the endothelial glycocalyx would be depicted. However, when the intravascular glycocalyx was injured by LPS administration, lanthanum nitrate particles were seen outside the vessel, implying blood flow out of the vessels. This may represent vascular permeability at the electron microscopic level. Endothelial glycocalyx injury promotes vascular permeability. However, whether lanthanum nitrate leaks out of the blood vessel, lanthanum nitrate leaks out and binds lanthanum nitrate to glycoproteins outside the blood vessel, or whether the endothelial glycocalyx to which lanthanum nitrate is bound to leaks out of the blood vessel is a subject for future research.

### 4.3 rhTM accelerates of Glypican-1 synthesis

Proteoglycans, which are the main components of the vascular endothelial glycocalyx, are the most important "backbone" molecules of the glycocalyx. Heparan sulfate proteoglycans account for approximately 50–90% of the total proteoglycans in the vascular endothelial glycocalyx, and glypican-1 is a heparan sulfate proteoglycan [32, 33].

Glypicans and syndecans are the two major families of heparan sulfate proteoglycans. Unlike syndecans, heparan sulfate glycosaminoglycan chains bound to glypicans are located close to the cell membrane. Six types of glypicans have been identified in mammals and are called GPC1–GPC6. Of the proteoglycans on the vascular endothelium, only two syndecans and glypicans bind to the cell membrane via the transmembrane domain and glycosylphosphatidylinositol anchor, respectively. Glypican family members are mainly distributed on the apical cell membrane but are also expressed in a cell type-specific manner, and only glypican-1 is present in vascular endothelial cells [34].

Since endothelial glycocalyx is synthesized by endothelial cells [35], the promoted expression of GPC-1 on endothelial cells is assumed to be closely related to endothelial glycocalyx synthesis under septic conditions.

Osteoglycine (also called mimecan), encoded by the *OGN* gene, is a secreted soluble proteoglycan that constitutes the vascular endothelial glycocalyx, and its expression is enhanced by rhTM. Thus, rhTM may promote the synthesis of proteoglycans that constitute the vascular endothelial glycocalyx in the heart under vasculitis-induced conditions, suggesting that rhTM contributes to protecting the vascular endothelial glycocalyx.

rhTM promotes the expression of HS6ST1, a synthetic enzyme of heparan sulfate, in the LPS-induced vasculitis model in the lungs [22], while the present study suggests that it promotes the synthesis of glypican, which is present in the vascular endothelium. Although both the cardiac and pulmonary capillaries take the form of continuous capillaries, the thickness of the vascular endothelial glycocalyx is different, and it can be assumed that the components of the vascular endothelial glycocalyx are also different [14]. Therefore, rhTM may act differently in the heart and lungs, although both molecules are involved in heparan sulfate proteoglycans.

### 4.4 Study limitations

Sepsis is a very complex disease than simple endotoxemia in experimental models. Since this study focused on investigating the direct relationship between endothelial glycocalyx damage and sepsis-induced vascular injury, we used an endotoxemia model that does not reflect typical septic conditions such as bacterial infection. This is a limitation of this study. In addition, the dosage of rhTM in this study was high at 30 mg/kg because (1) the rhTM used was a human recombinant preparation, not for mice, and (2) the route of administration was intraperitoneal, not intravenous or continuous, unlike in clinical use. Likewise, the extent of redistribution of rhTM from the peritoneum to the blood was not confirmed.

Although there was increased mortality with increased thrombomodulin in patients with sepsis and shedding of thrombomodulin in the previous report [36], the current study did not confirm whether rhTM interacted with soluble shed thrombomodulin.

## 5 Conclusions

Our results suggest that rhTM may improve cardiac microcirculation by inhibiting endothelial glycocalyx damage, suppressing inflammatory cytokine secretion, and enhancing glypican expression in heparan sulfate proteoglycans. Since rhTM has already been applied clinically, it can be considered a new strategy against sepsis-induced vascular injury via endothelial glycocalyx protection.

## Declarations

### Author contribution statement

Hideshi Okada, Tomoaki Doi – Conceived and designed the experiments; Analyzed and interpreted the data and Wrote the paper.

Yoshinori Kakino – Conceived and designed the experiments; Performed the experiments and Wrote the paper.

Hirotsugu Fukuda, Ayane Nishio, Y. Kawasaki, Ayumi Kuroda, Takuto Shimada, Yugo Wakayama, Tomoki Okuda, Haruka Okamoto, Genki Yoshimura, Ryo Kamidani, Ryu Yasuda, Tetsuya Fukuta, Yuichiro Kitagawa, Takahito Miyake and Shozo Yoshida – Performed the experiments.

Chihiro Takada – Analyzed and interpreted the data and contributed reagents, materials, analysis tools or data.

Nobuyuki Tetsuka, Hiroyuki Tomita, Keiko Suzuki, Shigeo Takashima and Shinji Ogura – Analyzed and interpreted the data.

Kodai Suzuki – Contributed reagents, materials, analysis tools or data.

### Funding statement

Shozo Yoshida was supported by Japan Society for the Promotion of Science [21K16570 & 18K16511].

Dr. Hideshi Okada was supported by Japan Society for the Promotion of Science [19H03756 & 16H05497].

Shinji Ogura, Ryu Yasuda, Haruka Okamoto, Yuichiro Kitagawa, Yoshinori Kakino, Tetsuya Fukuta, Tomoaki Doi, Takahiro Yoshida were supported by Japan Society for the Promotion of Science [21K09068, 20K17888, 20K17887, 20K17857, 20K17856, 19K18347, 19K09410, 18K08884].

### Data availability statement

Data associated with this study has been deposited at the Gene Expression Omnibus (GEO) under the accession number GSE126725 (http://www.ncbi.nlm.nih.gov/geo/).

### Declaration of interest’s statement

The authors declare no conflict of interest.

### Additional information

No additional information is available for this paper.

## Appendix A Supplementary data

Supplementary Figure.tif Ultrastructural Image of injured cardiac capillary in heart using a transmitted electron microscope. (A) Injured cardiac capillary was detected 48 h after LPS injection in the heart. (B and C) The expanded images from the white squares of (B) and (C) in (A). Arrows indicate capillary injury sites. Supplementary Figure.tif
