# Supplementary figures and images for: Recombinant thrombomodulin may protect cardiac capillary endothelial glycocalyx through promoting Glypican-1 expression under experimental endotoxemia
Source: Heliyon. 2022 Oct 25;8(11):e11262. doi: 10.1016/j.heliyon.2022.e11262 (PMC9637643; doi:10.1016/j.heliyon.2022.e11262)

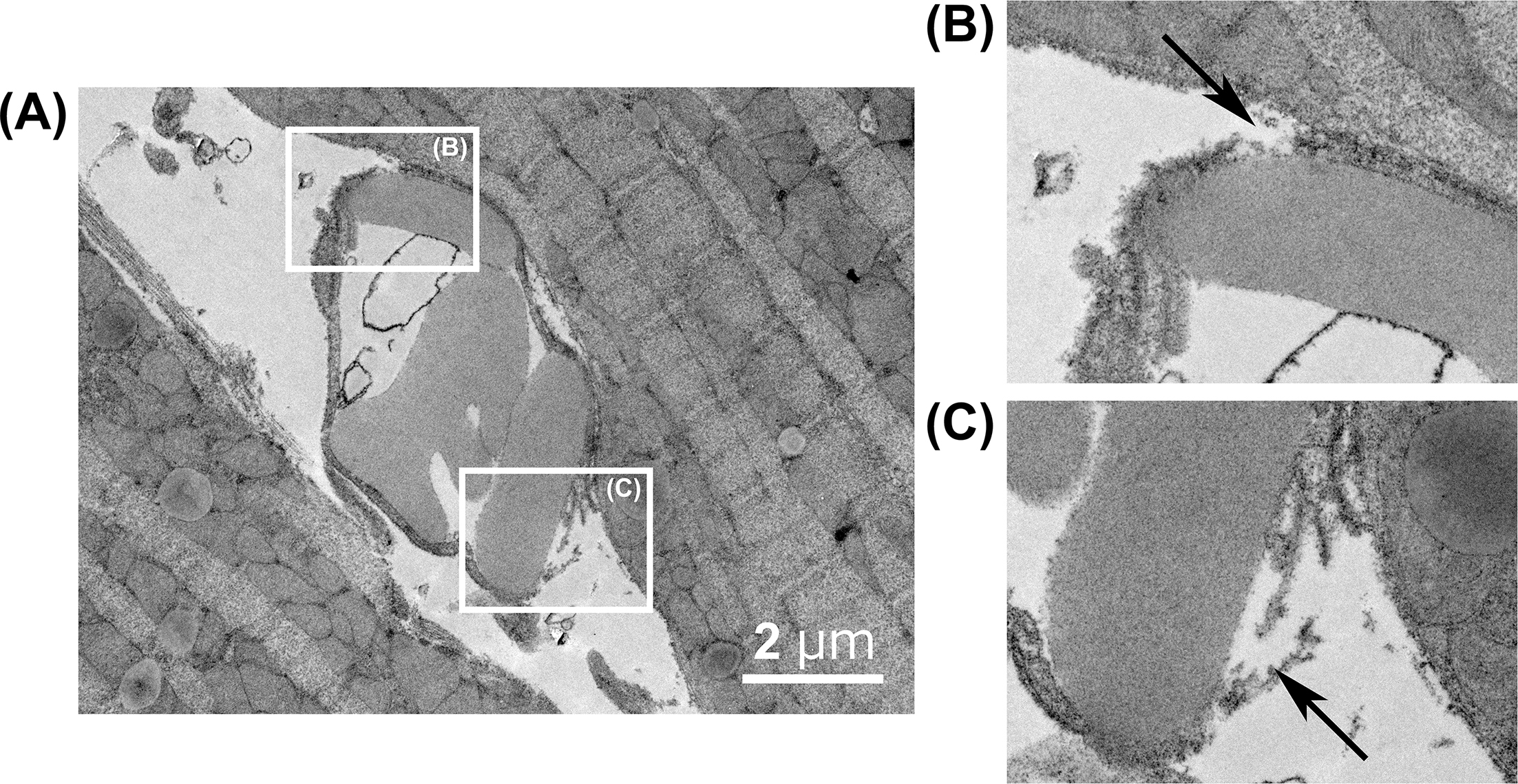

Supplement: Supplementary Figure.tif — Ultrastructural Image of injured cardiac capillary in heart using a transmitted electron microscope. (A) Injured cardiac capillary was detected 48 h after LPS injection in the heart. (B and C) The expanded images from the white squares of (B) and (C) in (A). Arrows indicate capillary injury sites. [file figs1.jpg]
